# Chemically programmable metastable solids: processing-history control of structure and reactivity in MXenes

**DOI:** 10.3389/fchem.2026.1912356

**Published:** 2026-08-19

**Authors:** Haotian Wu, Wenyue Si, Jiawen Tian, Bangsheng Yin

**Affiliations:** 1 College of Mechanical Engineering, Shandong Huayu University of Technology, Dezhou, Shandong, China; 2 Faculty of Mechanical and Automotive Engineering Technology, Universiti Malaysia Pahang Al-Sultan Abdullah, Pekan, Malaysia

**Keywords:** defects, intercalation, MXene, phase transformation, selective etching, solid-state chemistry, structure-property relation, surface termination

## Abstract

MXenes are commonly described by the compact formula M_n+1_X_n_T_x_, yet this notation hides the variables that most strongly determine their behavior: precursor ordering, extraction pathway, vacancies, termination identity and distribution, interlayer ions and water, stacking, and transformation history. This mini review develops a solid-state chemistry framework in which MXenes are treated as chemically programmable metastable solids. The central argument is that synthesis does not simply reveal a pre-existing two-dimensional carbide or nitride sheet; it drives the material along a coupled reaction coordinate that creates a history-dependent lattice, surface, and interlayer state. We connect precursor crystal chemistry to selective extraction, show why terminations and defects must be considered together, interpret intercalation as a structural component of the three-dimensional stacked solid, and examine oxidation and thermal conversion as mechanistic probes of metastability. Recent operando, spectroscopic, computational, and synthesis studies are integrated to identify transferable structure-property relations. We finally propose a minimum state descriptor and experimental priorities for converting empirical recipes into predictive synthesis. This perspective places MXenes squarely within solid-state chemistry and clarifies why nominally identical compositions can display divergent conductivity, mechanics, ion storage, and reactivity.

## 1 Introduction

MXenes are two-dimensional transition-metal carbides, nitrides, and carbonitrides typically produced by selectively removing an A-element layer from a layered precursor (Naguib et al., 2011; Naguib et al., 2012). Their compositional breadth, metallic or semiconducting electronic structures, hydrophilic surfaces, and ion-accessible galleries have supported applications ranging from electrochemical storage to catalysis, sensing, shielding, and mechanically adaptive composites. This application diversity has encouraged a convenient view in which a chosen M_n+1_X_n_ backbone supplies intrinsic electronic properties while the generic T_x_ suffix represents a tunable surface. This view is useful for classification, but it is chemically incomplete. Two samples both labeled Ti_3_C_2_T_x_ may differ in metal vacancies, residual Al, O/OH/F ratios, termination sites, trapped cations, interlayer water, flake thickness, turbostratic disorder, oxidation products, and strain. These differences are not secondary impurities around an otherwise fixed material. Together, they define the actual solid state.

The literature clearly illustrates this reproducibility problem. Processing guidelines for Ti_3_C_2_T_x_ showed that etchant composition, temperature, washing, delamination, and storage alter yield and material quality (Alhabeb et al., 2017). Nuclear magnetic resonance and multimodal spectroscopy further showed that terminations occupy distinct local environments and evolve during processing, rather than forming a compositionally featureless coating (Hart et al., 2021; Hope et al., 2016). Ion-exchange studies showed that the gallery contains specifically solvated cations whose identities affect spacing and electrochemical response (Ghidiu et al., 2016), while conductivity, friction, and failure vary with termination chemistry (Khanal and Irle, 2023; Yang et al., 2023). A nominal formula therefore cannot serve as a complete structure descriptor or a reliable basis for cross-study comparison.

**FIGURE 1:**
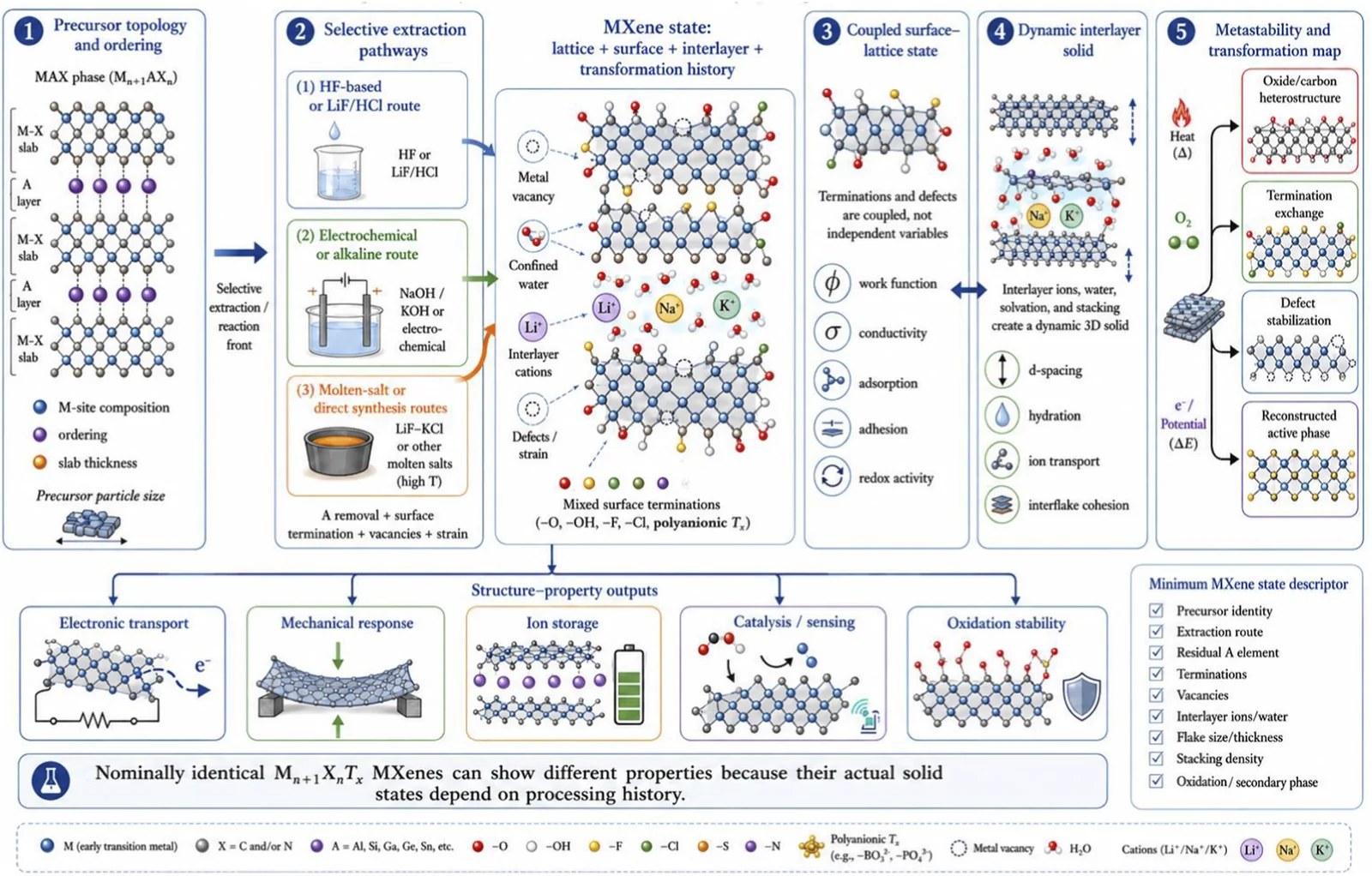
Processing-history reaction coordinate for chemically programmable metastable MXenes. Precursor topology and ordering constrain the accessible lattice; selective extraction co-produces the backbone, terminations, vacancies, strain, and interlayer species; and the resulting coupled surface-lattice and dynamic gallery states govern electronic transport, mechanical response, ion storage, catalysis/sensing, and oxidation stability. Thermal, chemical, and electrochemical driving forces can subsequently move the material among termination-exchanged, defect-stabilized, reconstructed, and oxide/carbon-derived states. The minimum state descriptor emphasizes that processing variables should be reported as structural information for reproducible structure-property attribution.

This review develops a solid-state reaction-coordinate framework. Here, the term reaction-coordinate framework does not denote a single universal kinetic equation. It denotes an ordered set of coupled state variables that evolve during precursor conversion, extraction, washing, delamination, post-treatment, and operation. An MXene state can be expressed conceptually as S = {B, V, T, G, W, K, X}, where B represents backbone composition and ordering, V vacancies, T termination identity and arrangement, G gallery ions or molecules, W hydration state, K stacking texture, and X transformation extent. A measured property can then be interpreted as P = f(S), rather than as a function of nominal composition alone. The accessible MXene state is first bounded by the topology, chemical ordering, and bonding of its precursor. Selective extraction then generates surfaces, vacancies, strain, and terminations through a coupled process. Washing and delamination reorganize that state through ion exchange, hydration, and stacking. Post-synthetic treatments can substitute terminations, stabilize defects, or drive conversion to carbides, oxides, carbon-supported phases, and heterostructures. Measurable function therefore emerges from the entire processing pathway rather than from the nominal composition alone. Accordingly, the central proposition is that processing history should be treated as a state variable: synthesis is not merely preparation before property measurement but part of the structure itself. Figure 1 summarizes this processing-history reaction coordinate and illustrates how precursor identity, extraction chemistry, coupled surface-lattice states, dynamic interlayers, and subsequent transformations jointly determine measurable structure-property outputs.

For readers less familiar with MXene chemistry, Figure 1 should be read from left to right as a sequence of state selection. The precursor panel defines which M–X backbone and ordering patterns can be inherited. The extraction panel then shows that A-layer removal is coupled with surface termination, vacancy formation, strain, and interlayer insertion rather than being a simple subtraction reaction. The central MXene-state panel represents the combined lattice, surface, gallery, and transformation history that is actually measured in experiments. The right-side panels emphasize that the interlayer region is a chemically active part of the stacked solid and that thermal, chemical, or electrochemical driving forces can move MXenes into termination-exchanged, defect-stabilized, reconstructed, or oxide/carbon-derived states. The bottom panels connect these state variables to electronic, mechanical, electrochemical, catalytic/sensing, and oxidation-related responses.

This framework is firmly grounded in solid-state chemistry because it emphasizes bonding in solids, order-disorder, reactivity of layered compounds, solid-liquid interfaces, phase transformations, and structure-property relations. Applications are considered only when they illuminate these relations. The central questions are as follows: Which lattice states are inherited from precursors? Which are generated kinetically during extraction? How do surface, defect, and interlayer variables couple? Which measurements can distinguish them? Answering these questions provides a more predictive foundation for MXene chemistry than cataloguing compositions or devices.

Unlike application-centered MXene reviews that organize the field by devices, performance metrics, or individual material systems, this mini review organizes MXene chemistry by state-forming reactions. The focus is not simply which MXene composition performs best, but which lattice, surface, interlayer, and transformation states are created by a given processing history. This distinction allows processing variables to be treated as structural information rather than experimental background, thereby providing a clearer basis for reproducible structure-property attribution.

The novelty of this perspective is not the isolated observation that processing affects MXene properties, which has been clearly established by foundational studies. Rather, the contribution is to organize these effects into a state-forming framework in which precursor topology, extraction-front chemistry, termination–defect coupling, gallery solvation, stacking texture, and transformation history are treated as coupled variables that define the actual MXene solid state. This framework converts processing history from a set of experimental details into structural information that can be used to formulate testable hypotheses, compare nominally identical samples, and identify which state variable is responsible for a measured property.

Previous MXene reviews have provided comprehensive summaries of synthesis methods, surface terminations, energy-storage behavior, catalytic activity, sensing performance, electromagnetic shielding, and composite architectures (Naguib et al., 2021; Liu et al., 2026). These reviews have been essential for mapping the breadth of MXene chemistry and applications. The present mini review differs in emphasis: it does not organize the field by application category or by isolated material composition, but by the sequence of state-forming reactions that convert a precursor into a history-dependent solid. In this sense, processing history is treated as a structural coordinate for interpreting reproducibility, metastability, and structure–property attribution, rather than as background information accompanying synthesis.

## 2 Precursor topology and selective extraction define the accessible lattice

The parent phase provides the first boundary condition. In conventional MAX phases, M-X slabs alternate with A-element planes, although the strength and distribution of M-A and M-X bonding vary with composition, layer thickness, alloying, and chemical order. Selective removal is possible because the A sublattice is generally more reactive, yet extraction must proceed without destroying the M-X framework. Consequently, etching selectivity reflects a competition among bond cleavage, diffusion of reactants and products, surface passivation, and mechanical relaxation. The resulting sheet is therefore not simply the parent slab minus A atoms; it is a reconstructed product whose surfaces and defects record the reaction.

Precursor design extends the accessible lattice beyond simple unary M sites. Mo_4_VAlC_4_ enabled a five-transition-metal-layer Mo_4_VC_4_ MXene (Deysher et al., 2019). Solid-solution approaches produced Nb-based M_4_C_3_ phases and continuously adjustable electronic structures (Yang et al., 2015), while controlled mixing of transition metals tuned optical and electronic properties (Han et al., 2020). In-plane ordering can have even greater consequences. Selective removal of both Al and Sc from an ordered parent yielded Mo_1.33_C with ordered divacancies (Tao et al., 2017), converting precursor order into a two-dimensional vacancy superstructure. These examples support a general principle: precursor symmetry and site occupancy are synthetic information that can survive, be amplified, or be edited during conversion.

Etchant chemistry determines the reaction pathway. Fluoride-containing aqueous routes couple A-layer dissolution with proton transfer, hydration, cation intercalation, and formation of O-, OH-, and F-containing surfaces. Small changes in acid concentration or LiF content alter reaction rate, residual precursor, flake size, and delamination behavior (Alhabeb et al., 2017; Ghidiu et al., 2014). Electrochemical etching provides a distinct, potential-biased reaction environment that has recently yielded both Ti_3_C_2_ and Ti_3_CN (Chan et al., 2024). However, potential control does not by itself guarantee complete A-layer removal or oxide-free surfaces; electrochemical routes can leave significant residual Al and introduce surface oxidation or mixed termination states. These limitations make mass balance, residual-A analysis, and surface-state characterization essential when electrochemical products are compared with fluoride-etched MXenes. Hydrothermal alkaline synthesis similarly changes the dissolution and termination landscape (Kulkarni et al., 2024). One-pot and air-compatible routes demonstrate that selective conversion can be accelerated, but rapid formation does not eliminate the need to resolve defect density and surface composition (Ma et al., 2021). In each case, the apparent endpoint of “MXene formation” can represent multiple microscopic endpoints.

Molten salts broaden the available chemistry by replacing proton-rich aqueous media with ionic reaction environments (Li et al., 2019). They can mediate Lewis-acid extraction, galvanic replacement, or electrochemical conversion while installing halogen or other terminations. Their significance extends beyond avoiding HF. Molten salts decouple some steps that are inseparable in water and allow reaction temperature, redox potential, and anion activity to be varied over wider ranges. Controlled or predominantly uniform halogen termination has been reported through multiphase synthesis (Li et al., 2026), and Lewis-basic halides can simultaneously tune termination and interlayer spacing (Zhang et al., 2022). These results show that the etchant also serves as a source of lattice-adjacent species and therefore a reagent for selecting solid states.

Mechanistically, selective extraction is best described as a moving reaction front. Local removal of A changes charge distribution and weakens adjacent layers; penetrant ions and solvent subsequently enter, terminations passivate exposed M atoms, and gas or soluble complexes leave. Unequal rates generate concentration gradients, pits, edge recession, residual A-rich regions, and stress. *In situ* or operando diffraction can distinguish uniform c-axis expansion from coexistence of parent, intermediate, and product phases, while microscopy can identify front propagation and vacancy clustering. Emerging non-aqueous and dry-extraction concepts may further extend this logic by matching extraction products with precursor thermodynamics, although their generality across MXene families still requires systematic validation. In practice, phase purity should encompass spatial and termination homogeneity, not only disappearance of parent reflections.

The precursor-to-MXene transformation therefore defines the envelope of accessible states. A subsequent treatment cannot necessarily repair disorder introduced at this stage, and an apparently beneficial vacancy concentration may be inseparable from altered terminations or residual ions. Comparisons among synthesis routes must control precursor batch, particle size, ordering, and reaction extent. Without such controls, property differences assigned to “surface chemistry” may actually originate from different inherited lattices.

## 3 Terminations and defects form a coupled surface-lattice state

Surface terminations are often described as independent substituents attached to an invariant carbide sheet. However, the terminal atom, adsorption site, coverage, local order, and neighboring vacancies jointly determine bonding and charge. Common aqueous MXene surfaces are dominated by O-, OH-, and F-containing terminations, whereas Cl and other halogen terminations are typically associated with specialized molten-salt or halide-mediated syntheses. These terminations can modify the transition-metal d states, work function, magnetic exchange, adsorption energy, and interlayer interactions. Calculations and experiments have shown that electronic properties can be controlled through termination and intercalation (Hart et al., 2019), that surface engineering changes magnetic anisotropy (Frey et al., 2019), and that solid-solution composition provides an additional, coupled electronic variable (Han et al., 2020). The chemically relevant object is therefore a surface-lattice configuration, not a list of functional groups.

The first challenge concerns measurement. X-ray photoelectron spectroscopy is sensitive to chemical states but peak assignments may be ambiguous when oxides, oxyfluorides, adsorbed water, and multiple termination sites coexist. NMR resolves local bonding environments and revealed heterogeneous Ti_3_C_2_ surface functionalization (Hope et al., 2016). Multimodal analysis of Cr_2_TiC_2_T_x_ demonstrated the need for complementary spectroscopies to follow termination evolution (Hart et al., 2021). Diffraction reports average spacing rather than termination identity, whereas electron microscopy can locate heavier atoms but may alter beam-sensitive surfaces. Reliable assignment therefore requires composition, local bonding, and structural measurements on the same batch. Reporting only an O:F atomic ratio is insufficient because the detected oxygen may originate from terminations, water, hydroxyls, or oxide domains.

The second challenge is the mutual compensation between defects and terminations. Metal or carbon vacancies change local coordination and create high-energy sites that bind terminations or intercalants more strongly. Conversely, the chemical potential imposed by an etchant can stabilize particular vacancy charge states. Ordered divacancies in Mo_1.33_C are inherited from the in-plane chemical ordering of the parent laminate after selective removal of Al and Sc, rather than being randomly generated during MXene synthesis (Tao et al., 2017). In contrast, disordered vacancies can arise from over-etching, oxidation, or other non-uniform reaction pathways. Recent work demonstrated that alkali cations stabilize defects at ambient and elevated temperatures (Wyatt et al., 2024), directly connecting vacancy persistence to interlayer species. Vanadium-deficient engineering and vacancy manipulation in V- and Mo-based MXenes similarly suggest that defects can enhance ion-storage reactions, but the mechanistic assignment must distinguish additional active sites from changes in spacing, conductivity, and termination coverage.

Termination substitution offers a route to controlled state changes, including covalent replacement and elimination reactions that access surface chemistries unavailable from routine aqueous etching (Kamysbayev et al., 2020). Predominantly halogen-terminated MXenes reported from specialized multiphase or molten-salt-related syntheses reduce some ambiguity associated with mixed aqueous O/OH/F surfaces (Li et al., 2026). Lewis-basic halides tune both interlayer separation and terminal groups (Zhang et al., 2022), demonstrating that these variables cannot always be changed independently. Surface modification with silanes or polymeric ligands can produce stronger interflake coupling and altered dispersion (Riazi et al., 2020), but covalent modification also changes the electronic and solvation environment. The most informative experiments therefore compare states connected by a chemically explicit reaction while measuring mass balance and lattice integrity.

Property trends further illustrate this coupling. Conductivity depends on carrier density and scattering from terminations, vacancies, adsorbates, and interflake junctions; the reported effect of functional groups (Khanal and Irle, 2023) cannot be generalized unless these other factors are specified. Friction and fracture reflect both intrinsic bond strength and interlayer adhesion, and termination-dependent changes can shift the balance between sliding and failure (Yang et al., 2023). In electrochemistry, termination redox, ion adsorption, and changes in the M oxidation state may overlap. Pseudo-intercalation in acidic electrolyte (Mu et al., 2019) and termination-dependent charge storage (Bo et al., 2025; Hart et al., 2019) demonstrate that assigning capacity to a single “active site” is misleading when protonation, solvation, and electronic screening change together.

A useful conceptual approach is to treat surface composition as an order parameter with short-range correlations, meaning that mixed terminations should not be assumed to be randomly distributed even when their average elemental ratios are similar. Local electrostatics, lattice registry, and synthesis gradients can favor domains or paired arrangements. Such organization could explain why samples with similar bulk elemental ratios can differ in work function or ion affinity. Establishing this picture requires pair-sensitive measurements, atomistic models with realistic disorder, and synthesis routes that vary chemical potentials systematically. The objective is not to identify a universally ideal termination, but to establish a reproducible map linking termination configuration and defect topology to a bounded property range.

A critical limitation of the current literature is that termination, defect density, residual A content, and oxidation are often measured separately or reported with different levels of uncertainty. As a result, trends assigned to a single termination species may partly reflect unresolved vacancy compensation, interlayer ions, or oxide formation. Future studies should therefore prioritize state-connected experiments, in which termination exchange, defect stabilization, and property measurement are performed on the same parent batch.

## 4 Interlayer guests, stacking, and solvation create a dynamic three-dimensional solid

Although an isolated monolayer provides a useful model, most measured MXenes are multilayer particles, films, membranes, or restacked electrodes. The resulting macroscopic solid is three-dimensional: two-dimensional sheets are coupled through ions, molecules, water, and registry-dependent forces. The gallery is therefore an integral component of the crystal chemistry. Ion-exchange and cation-solvation reactions in Ti_3_C_2_ demonstrated that interlayer spacing depends on both cation identity and solvation shell (Ghidiu et al., 2016). Termination and intercalation jointly control electronic properties (Hart et al., 2019), while Lewis-basic halides can change spacing and surface composition in one process (Zhang et al., 2022). Treating spacing solely as a geometric parameter obscures the chemical origin of the expanded lattice.

Interlayer water is particularly important because it acts as solvent, ligand, proton-transfer medium, and mechanical spacer. Its amount and configuration respond to humidity, termination basicity, cation charge density, and confinement. Water removal may contract the lattice and, under controlled conditions, improve interflake contact. However, strong vacuum drying or excessive dehydration of Ti_3_C_2_T_x_ can also cause gallery collapse, irreversible restacking, loss of electrostatic or solvation screening, and increased contact resistance, which may decrease rather than improve film conductivity. Conversely, excess water can facilitate swelling and ion transport but can accelerate oxidation. A reported d spacing is meaningful only when accompanied by hydration and environmental conditions. This is one reason *ex situ* diffraction can disagree with electrochemical behavior measured under bias.

Desolvation offers a mechanistic bridge between solution chemistry and solid-state insertion. Recent work demonstrated that controlling ion desolvation boosts charge storage in Ti_3_C_2_ (Bo et al., 2025). The energy cost is distributed across loss of bulk solvation, coordination by terminations, confinement, and electronic compensation in the sheet. Different cations can therefore produce similar spacings but different kinetics, or different spacings but similar charge transfer. Operando diffraction combined with spectroscopy and electrochemical analysis is required to distinguish adsorption at outer surfaces, insertion into galleries, termination redox, and deeper electronic changes.

Stacking order introduces an additional variable. Aligned flakes maximize in-plane conduction but create tortuous cross-plane transport; turbostratic disorder and pores shorten diffusion paths but increase junction resistance and expose edges. Mechanical processing, flake-size selection, and additives determine registry and texture. The strong performance of engineered hydrogels, aerogels, fibers, and gradient composites (Liu H. et al., 2022; Liu L.-X. et al., 2022; Wang et al., 2021; Wu et al., 2023; Xu et al., 2023; Xue et al., 2023) is relevant here not as an application catalogue, but as evidence that mesoscale assembly can dominate the properties attributed to the MXene chemistry. Heterostructures such as MoS_2_-on-MXene (Chen et al., 2018) and graphene-intercalated architectures (Wang G. et al., 2023) further demonstrate that an inserted solid changes strain, charge transfer, and gallery accessibility simultaneously. These examples are most relevant to the present framework when they reveal how gallery chemistry and stacking texture translate atomic-scale MXene states into macroscopic transport or mechanical responses.

Accordingly, the chemically meaningful hierarchy comprises monolayer lattice, termination/defect surface, solvated gallery, and mesoscale stack. Measurements should identify which level controls the reported response. For example, intrinsic calculations may predict a high conductivity for a termination state, whereas film conductivity is limited by oxidation and junctions. An ion-storage mechanism inferred from a bulk electrode can reflect pore architecture rather than lattice intercalation. Conversely, assembly is not purely extrinsic: confined ions and molecules can stabilize defects (Wyatt et al., 2024), alter termination equilibria, and select stacking arrangements. The hierarchy is coupled in both directions.

The main challenge is that many studies report interlayer spacing, conductivity, or ion-storage performance without independently resolving hydration, cation identity, and stacking texture. Such data are useful for performance comparison but insufficient for mechanistic attribution. The more critical question is not whether an expanded gallery improves a property, but which chemical species creates the expansion and whether the same state persists under the measurement environment.

## 5 Transformation chemistry exposes MXene metastability

MXenes are frequently called stable when they retain a diffraction pattern or device function for a chosen period. Solid-state chemistry requires a more precise question: stable against which reaction, under which chemical potentials, and on what timescale? The high surface area and under-coordinated metal sites that enable reactivity also favor oxidation, hydrolysis, termination loss, sintering, or conversion. In this review, metastability is used in an operational solid-state chemistry sense: many MXenes can persist for experimentally relevant timescales under selected environments, while remaining susceptible to oxidation, hydrolysis, termination loss, or conversion when chemical potentials change. Direct thermodynamic quantities such as formation enthalpy, entropy, or free energy are still unavailable for many experimentally relevant terminated and hydrated MXene states; therefore, transformation pathways and degradation products are treated here as mechanistic indicators rather than complete thermodynamic proof. Importantly, metastability should not be viewed only as a degradation liability. It also provides the chemical driving force for termination exchange, defect stabilization, controlled oxidation, and conversion into derived active phases.

Oxidation commonly nucleates at edges, defects, and locally exposed metal sites, then advances through the sheet. Early work converted MXenes into nanocrystalline transition-metal oxides supported on disordered carbon (Naguib et al., 2014), demonstrating that degradation can be redirected toward a synthetic transformation. Atomistic studies of Ti-based MXenes describe decomposition into carbon-supported Ti-O phases, with termination identity and temperature affecting reaction sequence. High-temperature experiments on Ti_3_C_2_T_x_ revealed a sequence of phase transformations rather than a single decomposition temperature (Wyatt et al., 2021). Composite environments can delay macroscopic oxidation (Liu N. et al., 2022), although stabilization may result from restricted oxygen transport, water management, radical scavenging, or altered heat transfer rather than stronger intrinsic M-X bonding.

Termination chemistry determines both the initial energy and the transformation pathway. O-rich surfaces may resemble an incipient oxide coordination environment, while halogen terminations can desorb or be exchanged before lattice oxidation. Mixed surfaces create local galvanic or strain heterogeneity. Water can accelerate conversion by solvating metal species and mediating proton transfer. Vacancy-rich regions can serve as nucleation sites, yet cation occupation may stabilize them (Wyatt et al., 2024). Accordingly, oxidation resistance should be reported together with termination composition, defect indicators, dissolved oxygen, light exposure, pH, temperature, and flake size.

Transformation need not be destructive. Controlled conversion can yield oxide/carbon heterostructures, catalytic interfaces, porous frameworks, or compositionally edited carbides. Mo_2_C sheets exhibit catalytic activity in CO_2_ hydrogenation (Zhou et al., 2021), while mild oxidation can create holey frameworks and additional redox sites. Emerging selective-lattice-editing and dry-extraction concepts may further extend this editing logic by removing or replacing selected lattice components, although their broader applicability remains to be established. These reactions therefore blur the boundary between MXene synthesis and post-MXene derivatization: a material may retain sheet morphology while its local bonding has moved beyond the original M_n+1_X_n_ lattice.

A transformation map provides an appropriate descriptor. Axes may include oxygen and water chemical potentials, temperature, electrochemical potential, and time; states include termination exchange, vacancy migration, interlayer collapse, oxide nucleation, carbon reorganization, and complete conversion. *In situ* diffraction tracks long-range phase evolution, Raman and infrared spectroscopy follow bonding, microscopy locates nucleation, and mass spectrometry identifies volatile products. Machine-learning-assisted tracking of structural evolution (Hollenbach et al., 2024) may help to integrate these signals, but models must be constrained by chemically identifiable intermediates rather than treating spectral changes as an abstract classification task.

Transformation maps also strengthen the interpretation of application data. A catalyst measured after cycling may be an oxyhydroxide or oxide derived from an MXene rather than the initial carbide. A battery electrode may undergo irreversible gallery reconstruction before reaching a steady response. The scientifically useful conclusion is not simply that the MXene “failed” or “remained stable,” but which state was active and how it formed. This distinction is central to connecting preparation, structure, properties, and reactivity.

## 6 From processing variables to transferable structure-property rules

The reaction-coordinate view helps explain why universal rankings of MXene compositions often fail. Consistent with the state-vector notation introduced above, a measured property can be written conceptually as P = f(S), where S includes backbone/order, vacancies, terminations, interlayer species, hydration, stacking texture, and transformation extent. This expression is not intended as a mathematical model, but as a reminder that these variables covary during processing. A more rigorous structure-property study therefore either holds the other variables constant or measures them sufficiently to model covariance. This state-vector formulation generates testable hypotheses: if a specific variable in S controls a property, then perturbing that variable within the same parent batch should change the measured response in a predictable direction, whereas holding that variable constant should reduce cross-sample variability.

Electronic transport illustrates this challenge. Backbone composition controls the available bands, but terminations shift orbital energies and carrier concentration (Han et al., 2020; Hart et al., 2019). Vacancies and oxidation introduce scattering or localization, water changes interflake separation, and film texture determines junction density. A conductivity value without density, orientation, humidity, and surface-state data cannot substantiate an intrinsic electronic trend. Mechanical behavior is likewise multiscale: termination-dependent adhesion affects sliding, defects concentrate stress, flake dimensions control crack bridging, and intercalants plasticize galleries (Yang et al., 2023). High toughness in fibers or hydrogels (Liu H. et al., 2022; Liu L.-X. et al., 2022) may therefore be genuine while revealing little about the intrinsic fracture resistance of a monolayer.

Electrochemical storage introduces time-dependent changes in state. Ion exchange, solvation, pseudocapacitive surface redox, and lattice electronic compensation can occur over overlapping potentials (Bo et al., 2025; Ghidiu et al., 2016; Mu et al., 2019). Apparent capacity may increase through vacancies or porosity, but the same modification may lower conductivity or accelerate conversion. The most transferable descriptor is not the maximum capacity, but a mechanism-resolved relation among ion coordination, structural change, charge distribution, and reversibility. Operando measurements of spacing alone are insufficient because swelling can occur without equivalent electron transfer; spectroscopy and quantitative charge balance are required.

Catalytic studies require verification of the active state. Surface terminations regulate adsorption energies and proton transfer, whereas defects and supported phases create distinct coordination environments. Mo_2_C catalysis (Zhou et al., 2021) and MXene-derived or MXene-supported chalcogenide systems (Zhang et al., 2024) demonstrate useful reactivity; however, post-reaction characterization is essential to determine whether activity belongs to the carbide surface, a reconstructed phase, or an interface. The same caution extends to gas sensing and electromagnetic response: changes in conductivity or polarization may be dominated by oxidation, adsorbed water, or heterointerfaces rather than the nominal MXene (Cai and Kim, 2025; Wang G. et al., 2023).

Table 1 organizes the principal control variables according to structural signature, mechanistic consequence, and diagnostic need. Rather than prescribing a single synthesis, it converts recipe variables into testable solid-state hypotheses. If a molten-salt product displays improved conductivity, the hypothesis should specify whether this arises from reduced F coverage, more uniform terminations, fewer hydrated junctions, or altered defect density. If alkali treatment improves thermal persistence, cation occupancy and vacancy migration should be measured (Wyatt et al., 2024). Each claimed improvement in properties should be linked to a resolved state change.

**TABLE 1:** Processing-history variables that define MXene solid states, their mechanistic consequences, and the evidence required for structure-property attribution.

Control lever	Defining structural or chemical variables	Mechanistic consequence	Priority diagnostics	Representative references
Precursor topology and ordering	M-site composition; in-plane/out-of-plane order; M-X slab thickness; precursor particle size	Bounds the accessible backbone, electronic structure, extraction selectivity, and inherited vacancy order	XRD; STEM-EDS; diffraction simulation; precursor particle-size analysis	Deysher et al. (2019), Han et al. (2020), Naguib et al. (2011), Naguib et al. (2012), Tao et al. (2017), Yang et al. (2015)
Aqueous or electrochemical selective extraction	Residual A element; reaction-front intermediates; pits; strain; flake-size change; mixed O/OH/F terminations	Couples A removal to reconstruction, defect formation, hydration, and delamination	Operando XRD; solution ICP; XPS; STEM; quantitative mass balance	Alhabeb et al. (2017), Chan et al. (2024), Ghidiu et al. (2014), Ma et al. (2021)
Molten-salt and non-aqueous/direct synthesis	Redox potential; Lewis acidity/basicity; halide activity; gas/solid precursors; growth morphology	Expands accessible precursors, terminations, and reaction environments; in direct routes, the backbone can form without conventional MAX-phase extraction	*In situ* XRD; thermal analysis; gas/product analysis; microscopy; termination spectroscopy	Li et al. (2019), Li et al. (2026), Wang et al. (2023a), Zhang et al. (2022)
Termination identity and arrangement	Common O/OH/F terminations; specialized halogen or covalent terminations; adsorption sites; coverage; short-range order	Changes d states, work function, magnetic exchange, adsorption, adhesion, and redox	NMR; XPS with standards; vibrational spectroscopy; STEM/EELS; DFT constrained by experiment	Frey et al. (2019), Hart et al. (2019), Hart et al. (2021), Hope et al. (2016), Kamysbayev et al. (2020), Li et al. (2026)
Vacancies and charge compensation	Ordered/disordered M or X vacancies; compensating cations; local strain	Creates active sites and scattering centers and modifies migration, mechanics, and thermal persistence	Atomic-resolution STEM; pair-distribution analysis; elemental analysis; temperature-dependent spectroscopy	Khanal and Irle (2023), Tao et al. (2017), Wyatt et al. (2024), Yang et al. (2023)
Interlayer ions, water, and molecules	Cation identity; solvation shell; hydration; gallery spacing; protonation state	Controls desolvation, ion transfer, screening, proton transfer, and interflake cohesion	Humidity-controlled/operando XRD; NMR; QCM; electrochemical spectroscopy	Bo et al. (2025), Ghidiu et al. (2014), Ghidiu et al. (2016), Mu et al. (2019), Zhang et al. (2022)
Stacking and mesoscale texture	Registry; alignment; porosity; junction density; heterointerfaces; film density	Governs cross-plane transport, mechanical response, and composite-level polarization	SAXS/WAXS; tomography; density; anisotropic transport; mechanical testing	Chen et al. (2018), Liu et al. (2022b), Wang et al. (2023b), Wu et al. (2023), Xu et al. (2023), Xue et al. (2023)
Thermal, oxidative, and reactive history	Termination loss; oxide nucleation; carbon reorganization; reconstructed or supported phases	Selects kinetic persistence, degradation pathway, or conversion into a new active solid	*In situ* XRD/Raman; TGA-MS; XPS depth profiling; post-reaction microscopy	Hollenbach et al. (2024), Liu et al. (2022c), Naguib et al. (2014), Wyatt et al. (2021), Wyatt et al. (2024), Zhou et al. (2021)

To improve practical reproducibility, we propose a minimum MXene state descriptor: (i) precursor identity, ordering, and particle size; (ii) extraction reagents, chemical potentials, temperature, and time; (iii) residual A content and vacancy indicators; (iv) termination identities with method-dependent uncertainty; (v) interlayer ions and water under defined humidity; (vi) flake thickness and lateral-size distribution; (vii) stacking texture and film density; and (viii) oxidation or secondary-phase fraction before and after measurement. Although this descriptor is longer than M_n+1_X_n_T_x_, it more closely captures the information required to reproduce a solid.

These descriptors were selected because they correspond to the main state variables identified throughout this review: precursor-defined backbone, extraction-generated defects and residues, surface termination chemistry, solvated gallery composition, flake morphology, stacking texture, and transformation extent. They are therefore intended to define a minimum reporting set for reproducibility and cross-study comparison, not a complete atomic-resolution description of every local configuration. Important limitations remain. The descriptor may not capture nanoscale termination ordering, buried interfacial chemistry, transient operando states, beam- or drying-induced changes, or spatial heterogeneity across large films and composites. Accordingly, the descriptor should be used together with method-dependent uncertainty and, when possible, perturbation experiments that test which variable actually controls the measured property.

### 6.1 Cross-scale evidence and attribution limits

Application studies provide some of the clearest evidence that processing history matters, yet they also present the greatest risk of assigning a composite-level effect to an unresolved atomic variable. This section does not review applications for performance comparison; instead, it uses selected application studies as stress tests for the proposed state-attribution framework. Gas sensors illustrate both aspects. Adsorption changes the carrier density and scattering of a conductive MXene, so termination identity, defect concentration, and water coverage should influence sensitivity and selectivity. However, a sensor signal also depends on film porosity, contacts, humidity, analyte diffusion, and progressive oxidation. The broad gas-sensing literature (Cai and Kim, 2025) therefore confirms the chemical importance of the surface while showing that performance tables cannot by themselves establish a termination-specific mechanism. A rigorous experiment would use one parent batch, exchange or modify a defined termination, equilibrate hydration, and compare adsorption spectroscopy with electrical response under identical film geometry.

The distinction between intrinsic and assembled responses is equally important in electromagnetic and mechanical systems. Three-dimensional printed gradient frames (Xue et al., 2023), hollow NiCo-containing architectures (Wang et al., 2021), MXene/carbon aerogels (Wu et al., 2023), nanocellulose-supported aerogels (Xu et al., 2023), and aramid-nanofiber coaxial fibers (Liu L.-X. et al., 2022) achieve properties that depend on multiple interfaces and length scales. These studies are informative because they reveal how sheet alignment, interfacial polarization, crack deflection, and percolation amplify the behavior of a chemically active building block. However, the same complexity prevents a direct inference that a measured shielding efficiency or toughness arose from a particular M-X backbone or termination. The appropriate solid-state conclusion is therefore conditional: a specified MXene state enables an assembly pathway, and that pathway creates the dominant macroscopic mechanism. Both links must be measured.

Hydrogel dynamics provide another example. Highly deformable MXene hybrid hydrogels can display rapid mechanical recovery and temperature-dependent response (Liu H. et al., 2022). Such behavior reflects polymer or supramolecular network relaxation, confined water, ion redistribution, and reversible interflake contacts. This environment may also alter the MXene state by changing local hydration and oxygen transport. Consequently, the host matrix is not a passive support. It changes chemical potentials around the sheets and can stabilize states that would not persist in a dry film. Comparisons between free flakes and embedded flakes should therefore include termination and oxidation analysis after processing, not only before incorporation.

Electrode composites present similar attribution challenges. A CoSe_2_/CNT-MXene anode combines conversion or alloying reactions, conductive pathways, and mechanically buffered interfaces (Xu et al., 2021). Improved cycling may arise from the MXene backbone, but may instead reflect dispersion of the active phase, suppression of aggregation, or formation of a new interphase. The same logic applies to supported catalysts and heterojunctions. Mechanistic claims should include controls that replace MXene with a conductivity-matched carbon, vary termination while preserving morphology, and characterize the post-cycling phase. Otherwise, “synergy” remains a label for unseparated variables.

Compositionally distinct MXenes also require careful interpretation. The synthesis and characterization of two-dimensional Mo_2_C (Halim et al., 2016) established that MXene chemistry extends beyond Ti_3_C_2_, but different metals change bond covalency, oxidation tendency, and preferred terminations. Insights from Ti-based aqueous synthesis cannot be directly transferred to Mo-, V-, Cr-, or Nb-containing systems. The decade-scale synthesis literature (Naguib et al., 2021) documents the rapid expansion of compositions and routes, but this diversity increases the need for family-specific reaction maps. A useful taxonomy should classify materials by chemically relevant variables such as M-A extraction thermodynamics, M-X framework stability, accessible oxidation states, and termination exchange kinetics, rather than assuming that all members respond similarly because they share the MXene label.

Direct synthesis and chemical vapor deposition have recently expanded access to selected carbide and nitride MXene sheets without requiring a layered MAX-phase precursor (Wang D. et al., 2023). This advance expands the accessible reaction coordinate: backbone formation, morphology development, and surface-state generation can occur together rather than through extraction of a preformed A sublattice. It also provides a stringent test of transferability. If directly synthesized and etched MXenes with comparable backbone composition behave differently, the difference can reveal inherited defects, termination distributions, stacking, or residual species that nominal formulas omit. These materials should therefore be compared using the same state descriptors rather than treated as equivalent solely because their M-X compositions match.

Together, these examples emphasize that MXene state variables, such as termination, hydration, vacancy density, or transformation extent, should be distinguished from architecture-level variables, including film porosity, filler geometry, network relaxation, and composite interfacial structure.

To make attribution more rigorous, cross-scale MXene studies should include an explicit attribution protocol. First, one parent MXene batch should be used whenever possible to avoid batch-to-batch variation in precursor residue, vacancy density, and flake size. Second, the variable of interest, such as termination identity, hydration state, interlayer cation, or stacking density, should be perturbed while other variables are held constant or measured. Third, morphology-matched and conductivity-matched controls, such as carbon films or non-MXene conductive fillers, should be included when composite-level transport or shielding is evaluated. Fourth, the same specimen should be characterized before, during, and after operation using surface-sensitive and structural probes. Finally, the active post-operation state should be reported, because the functional material may be a hydrated, oxidized, reconstructed, or composite-interfaced MXene state rather than the initially prepared sheet.

These cross-scale examples support a hierarchy of evidence. Correlation between a recipe and performance represents the first level. Correlation between a measured state variable and performance is stronger. Reversible perturbation of that variable within the same specimen provides still stronger evidence. The most compelling evidence combines reversible control, operando structural observation, and a quantitative model that predicts the direction and magnitude of response. Applying this hierarchy could recast many apparently contradictory application results into bounded statements about different states and scales.

## 7 Discussion and outlook

MXene research is moving beyond discovery by etching toward synthesis by state selection. The decisive advance will come not from another isolated preparation recipe, but from a predictive connection between precursor thermodynamics, reaction-front kinetics, surface chemical potentials, defect compensation, and gallery equilibria. Three priorities follow.

First, synthesis studies should resolve reaction pathways rather than comparing endpoints alone. Time-resolved diffraction, spectroscopy, solution analysis, and microscopy can reveal intermediate phases and rate-limiting steps. These measurements are particularly important for molten-salt, electrochemical, hydrothermal, and dry extraction routes, where reaction environments differ fundamentally from aqueous fluoride etching. Quantitative mass balance should accompany claims of selectivity, because disappearance of an A element from diffraction does not demonstrate its complete removal or the preservation of the M-X lattice.

Second, realistic structural models should replace ideal, uniformly terminated slabs in experimental interpretation. Models should include mixed terminations, vacancies, water, cations, and finite-temperature disorder at concentrations constrained by measurement. Theoretical predictions remain valuable for identifying general trends, but agreement obtained by choosing an ideal termination after the experiment does not constitute mechanistic validation. Iteration between pair-sensitive spectroscopy, microscopy, diffraction, and computation can constrain the ensemble of plausible states. Data-driven tracking (Hollenbach et al., 2024) is promising when its features are linked to chemically interpretable coordinates.

Third, property measurements should be designed as controlled perturbation experiments. Ion exchange, controlled humidity, termination substitution, annealing, and electrochemical potential can move a sample along a known coordinate. Tracking the same batch before, during, and after perturbation reduces uncertainty caused by independent synthesis. These experiments can distinguish causation from correlation and reveal reversibility. They also make disagreements productive: divergent results may map different regions of a state diagram rather than indicate experimental failure.

A practical validation sequence can be implemented without using every available technique. First, diffraction and elemental analysis should determine conversion extent, residual precursor, and average gallery structure. Second, at least two chemically complementary surface probes should distinguish terminations from oxide, adsorbed water, and molecular contamination. Third, a controlled perturbation such as humidity exchange, cation exchange, or mild annealing should determine whether the assigned state changes in the predicted direction. Fourth, the target property should be measured during or immediately after that perturbation, with an untreated aliquot from the same batch as the control. Finally, post-measurement characterization should establish whether the material returned to its initial state or entered a new phase. This sequence cannot resolve every local configuration, but it can reject many incorrect causal assignments. Shared reference materials and round-robin measurements could further reveal laboratory-dependent effects in washing, drying, storage, and spectral fitting. Publishing negative results, including unsuccessful termination exchange or loss of conductivity after purification, would be particularly valuable because they constrain the accessible-state envelope and expose hidden trade-offs that maximum-performance reports tend to omit.

Several challenges remain before such state-based comparison can become routine. First, reproducibility requires shared reference materials or at least well-documented parent MAX phases or other well-defined precursor batches, because precursor particle size, impurity phases, and ordering can bias all subsequent processing steps (Alhabeb et al., 2017; Naguib et al., 2021). Second, standardized characterization protocols are needed for residual A content, termination assignment, hydration level, oxidation fraction, and flake-size distribution (Ghidiu et al., 2016; Hart et al., 2021; Hope et al., 2016; Wyatt et al., 2021). At present, differences in washing, drying, storage humidity, XPS fitting, and film-density measurement can produce apparently contradictory results even for nominally identical Ti_3_C_2_T_x_ (Alhabeb et al., 2017; Ghidiu et al., 2016; Hart et al., 2019). Third, operando and post-operation measurements must be more widely integrated, because the active state during catalysis, sensing, or electrochemical cycling may differ from the as-prepared state (Bo et al., 2025; Hart et al., 2019; Hollenbach et al., 2024; Wyatt et al., 2021). Addressing these challenges will require not only advanced techniques, but also reporting standards that specify environmental history, uncertainty, and cross-laboratory calibration.

This framework also has important limitations. Comprehensive characterization of every batch is impractical, and some variables are altered by the measurement itself. Surface-sensitive methods may not represent buried galleries, electron beams can damage terminations, and drying can change hydration and stacking. The proposed descriptor should therefore include uncertainty and measurement conditions. Moreover, application-driven architectures remain valuable even when mesoscale assembly dominates; the aim is to avoid attributing their performance to an unresolved intrinsic MXene property.

In conclusion, MXenes are most usefully understood as chemically programmable metastable solids whose lattice, surface, and interlayer states are co-produced by processing. Precursor ordering determines what can be inherited; selective extraction creates a reconstructed and terminated lattice; ions, water, and stacking complete the three-dimensional solid; and thermal or chemical perturbations expose competing phases. This perspective explains why nominally identical MXenes can behave differently and offers a route toward reproducibility. By reporting processing history as structural information and using operando perturbations to connect state changes with function, the field can move from empirical optimization to predictive solid-state chemistry.
